# Elective ICP monitoring: how long is long enough?

**DOI:** 10.1186/2045-8118-12-S1-P52

**Published:** 2015-09-18

**Authors:** Simon Thompson, Hasan Asif, Claudia Craven, Patricia Haylock-Vize, Edward Dyson, Aswin Chari, Samir Matloob, Neekhil Patel, Syed Shah, Andrew Stevens, Huan Wee Chan, Jinendra Ekanayake, Ahmed Toma, Lewis Thorne, Laurence Watkins

**Affiliations:** 1 The National Hospital for Neurology and Neurosurgery, UK; 2 Imperial College London, UK

## Introduction

Elective intracranial pressure (ICP) monitoring is a useful tool in the diagnosis and evaluation of simple and complex cerebrospinal fluid dynamic disturbances. Whilst many previous research papers have focused on patients undergoing ICP monitoring acutely following traumatic brain injury (TBI), few have looked into the duration of monitoring required to achieve an accurate picture of a patients intracranial dynamics in non acute, elective cases. At our institution we currently complete monitoring for a period of >48hrs.

## Methods

A retrospective audit, assessing any patient admitted electively to our institution for ICP monitoring over a 3 month period. Exclusion criteria included acute admissions and patients who underwent a change in their treatment whilst undergoing ICP monitoring (such as CSF shunt valve adjustment / surgical procedures and/or medication changes which could affect ICP i.e. Acetazolamide). ICP results were analysed focusing on median ICP and Median pulse amplitude over three time periods: total data collected v first 48hrs of data collection v first 24hrs of data collection.

## Results

18 patients met the desired criteria. Mean length of monitoring was 3 days (range 2-5) for the total number of patients. There was no significant difference between 24hrs and 48hrs duration of monitoring for the median ICP (p=>0.05) and ICP pulse amplitude (p=>0.05).

## Conclusion

24 hour monitoring of ICP in elective patients in a stable condition without changes to their current treatment is sufficient to detect mean ICP and pulse amplitude. Further studies may be appropriate to assess if fewer than 24hrs monitoring can also prove an accurate method of monitoring ICP.

